# Use of Food Additive Titanium Dioxide (E171) before the Introduction of Regulatory Restrictions Due to Concern for Genotoxicity

**DOI:** 10.3390/foods10081910

**Published:** 2021-08-17

**Authors:** Urška Blaznik, Sanja Krušič, Maša Hribar, Anita Kušar, Katja Žmitek, Igor Pravst

**Affiliations:** 1 National Institute of Public Health, Trubarjeva 2, SI-1000 Ljubljana, Slovenia; urska.blaznik@nijz.si; 2 Nutrition Institute, Tržaška Cesta 40, SI-1000 Ljubljana, Slovenia; sanja.krusic@nutris.org (S.K.); masa.hribar@nutris.org (M.H.); anita.kusar@nutris.org (A.K.); katja.zmitek@vist.si (K.Ž.); 3 VIST—Higher School of Applied Sciences, Gerbičeva Cesta 51A, SI-1000 Ljubljana, Slovenia; 4 Biotechnical Faculty, University of Ljubljana, Jamnikarjeva 101, SI-1000 Ljubljana, Slovenia

**Keywords:** titanium dioxide, E171, food supply, nanoparticles, safety, Europe, Slovenia

## Abstract

Food-grade titanium dioxide (TiO_2_; E171) is a coloring food additive. In May 2021, a scientific opinion was published by the European Food Safety Authority concluding that TiO_2_ can no longer be considered as a safe food additive. Our aim was to investigate the trends in the use of TiO_2_ in the food supply. A case study was conducted in Slovenia using two nationally representative cross-sectional datasets of branded foods. Analysis was performed on *N* = 12,644 foods (6012 and 6632 in 2017 and 2020, respectively) from 15 food subcategories where TiO_2_ was found as a food additive. A significant decrease was observed in the use of TiO_2_ (3.6% vs. 1.8%; *p* < 0.01). TiO_2_ was most often used in the chewing gum category (36.3%) in 2017, and chocolate and sweets category (45.9%) in 2020. Meanwhile, in 2017, the largest share of TiO_2_-containing foods was observed in the chewing gum category, namely, 70.3%, and these products presented over 85% of the market share. In 2020, only 24.6% of chewing gums contained TiO_2_, which accounted for only 3% of the market share. In conclusion, we showed an overall decrease in TiO_2_ use, even though it has not yet been officially removed from the list of authorized food additives.

## 1. Introduction

Titanium dioxide (TiO_2_) is a transition metal oxide with application as a pigment or photocatalyst [1]. As a white pigment it has been added to a variety of food products, including bakery products, sauces, cheeses, edible ices and sweets. In addition to food, titanium dioxide is also used in medicinal products as an excipient, and in personal care products as a pigment and thickener [2,3], and can also be used as an UV filter in mineral sunscreen products [4,5].

TiO_2_ was first approved for use in food in 1966 by the US Food and Drug Administration (FDA), with the stipulation that its content must not exceed 1% of the food weight [6]. On the basis of the Codex Alimentarius of the Food and Agriculture Organization/World Health Organization (FAO/WHO) [1] safety evaluation, TiO_2_ has been authorized as a food additive by the European Union (EU) with code E171 since 1969 [7]. Due to the presence of a fraction of nanoparticles, it falls under the scope of the EFSA Guidance on nanotechnology as “a material that is not engineered as nanomaterial but contains a fraction of particles, less than 50% in the number–size distribution, with one or more external dimensions in the size range 1–100 nm” [8]. E171 as a food additive consist of approximately 40% of TiO_2_ nanosized particles (<100 nm) and 60% of TiO_2_ microsized particles (>100 nm) [2,9,10]. As it was permitted for use in the EU before 20 January 2009, it belongs to the group of food additives that are subject to a safety re-evaluation by the European Food Safety Authority (EFSA), according to Commission Regulation (EU) No. 257/2010, and in line with the provision of Regulation (EC) No. 1333/2008 [11]. Therefore, the safety of TiO_2_ as a food additive was re-evaluated by the EFSA Panel on Food Additives and Nutrient Sources added to Food (ANS) [12] in 2016, on the basis of which the EFSA concluded that TiO_2_ did not raise concerns with respect to genotoxicity and carcinogenicity. Genotoxicity refers to the ability of a chemical substance to damage the genetic material of cells, which may lead to carcinogenic effects [13]. EFSA also recommended that additional studies be conducted to fill the gaps in possible effects on the reproductive system, which could lead to an established Acceptable Daily Intake (ADI) for TiO_2_. Therefore, in January 2017, the European Commission (EC) issued an open call for additional data for TiO_2_, including reproductive toxicity data. Several studies investigated the toxicity of dietary TiO_2_ [10,14,15,16,17,18,19,20,21,22,23,24,25], raising some concerns regarding its potential tumor-promoting activity. In 2018, the outcome of four specific studies [10,14,20,23] was included in a scientific evaluation to determine the need to re-open the conclusion of the EFSA’s opinion from 2016. However, the decision was taken in 2018 that the re-opening of this issue was not needed [26]. In April 2019, the French Agency for Food, Environmental and Occupational Health and Safety (ANSES) delivered a scientific opinion, based on 25 studies published between 2017 and 2019 [27], on the exposure to nanoparticles of TiO_2_, and highlighted that the previous EFSA assessment did not consider all available data. In response to this opinion [28], the EFSA noted that ANSES reiterated previously identified concerns and data gaps, and did not present findings that changed the Authority’s previous conclusions on the safety of TiO_2_. Furthermore, the Office for Risk Assessment and Research of the Netherlands Food and Consumer Product Safety Authority (NVWA) delivered an opinion on possible health effects of TiO_2_ in 2019 [29], highlighting the possible immune and reproductive toxicological effects of TiO_2_. While further activities were underway to obtain new data, the French Government followed the precautionary principle, based on the opinion of the ANSES in 2019 [27], and decided to ban TiO_2_ in food products starting on 1 January 2020. Just a few days after this decision was announced, a joint letter to the EC [30] was published to EC, with civil society organizations requesting to remove TiO_2_ from the EU list of permitted food additives. Following the request of the EC in March 2020, the EFSA started an additional safety evaluation of this additive. An in-depth safety assessment report for the TiO_2_ was published on 6 May 2021 [31]. The EFSA panel concluded that with consideration of the available evidence, a concern for genotoxicity could not be excluded and, therefore, TiO_2_ could no longer be considered as a safe food additive.

As mentioned, several studies have addressed the question of toxicity of E171. Studies on rats and mice have shown that nanoparticles can pass through the intestinal barrier, accumulate in the intestine and cause preneoplastic lesions [14,32], promote anxiety, increase the number of adenomas in the colon, induce hypertrophy and hyperplasia in goblet cells [33] and disrupt gut microbiota composition and function [34,35,36,37,38]. Accumulation and toxic effects have also been found in plants [39,40]. However, health aspects of E171 oral intake by consumers in a real exposure environment still need to be confirmed by further research.

Food additives are an important part of processed foods. Consumers have expressed concern for some time about their possible adverse health effects [41] and would like to be better informed about their potential health implications [42,43]. EU Member States, and the EC as risk managers, request the EFSA to provide independent scientific advice, which informs European food policy makers. In the next step, the EFSA’s scientific advice on TiO_2_ will be used to support further regulatory procedures and decisions. The most realistic outcome is that the use of TiO_2_ as a food additive will not be approved in the EU in the near future.

The objective of this study was to evaluate the prevalence and changes in the use of TiO_2_ as a food additive in the food supply since 2017, when the EC issued an open call for additional toxicity data for TiO_2_. The Slovenian food supply was selected for a case study, using nationally representative cross-sectional data on the composition of prepacked foods in 2017 and 2020 collected within the national “Nutrition and Public Health” research program and the “Food Nutrition Security Cloud” project (FNS-Cloud; www.fns-cloud.eu, accessed: 15 August 2021).

## 2. Materials and Methods

### 2.1. Data Collection and Categorization

The study was conducted on a sample of prepacked foods available in Slovenia, EU. The food supply sample was collected in 2017 and 2020 in major retail shops representing the majority of the food market, and was part of the Composition and Labelling Information System (CLAS, Nutrition Institute, Ljubljana, Slovenia) [44]. In both years, data collection was done in retail shops of Mercator, Spar, Tuš, Lidl, Hofer, while in 2020 we also included retailer Eurospin. The dataset was prepared by the extraction of food labelling information from photographs of all branded foods available in selected food stores at the time of collection. Data were collected with the aim of monitoring the nutritional composition of processed foods in the food supply [45], with the adaptation that we also collected ingredient lists. The detailed methodology of the data collection is described elsewhere [46,47].

Foods were classified into food categories according to Global Food Monitoring Group (GFMG) recommendations [45], with minor modifications [46,47]. Without food supplements, food additives sold to consumers in food stores and food that did not fit into any of the GFMG food groups, our dataset contained 49,919 prepacked food items; 23,690 and 26,229 from 2017 and 2020 monitoring, respectively. For 10,034 products (42%) in the 2017 dataset, there was a matching product with same International/European Article Number (EAN) barcode in the 2020 dataset. We identified all foods in this dataset, where the ingredient list text contained the terms “TiO_2_”, “E171” and/or “titanium (di)oxide”.

Food (sub)categories that contained foods with TiO_2_ as a food additive at least in one sampled year and were further investigated in this study are as follows: biscuits; cakes, muffins and pastry; canned fish with vegetables; chewing gum; chocolate and sweets; cordials; desserts; flavored yogurt; ice cream and edible ices; jelly; processed fish products; side dishes; soup; spreads and processed cheese; and sugar. Our total study sample, therefore, included between 12.664 and 6.012 foods for 2017, of which 215 contained TiO_2_ (3.6%), and 6.632 foods for 2020, of which 122 (1.8%) contained TiO_2_.

### 2.2. Data Processing and Statistical Analyses

Food composition data were processed using Microsoft SQL Server Management Studio 13.0, Microsoft Analysis Services Client Tools 13.0, Microsoft Data Access Components (MDAC) 10.0, Microsoft Excel 2019 (Microsoft, Redmond, Washington, DC, USA) and the Composition and Labelling Information System (CLAS) (Nutrition Institute, Ljubljana, Slovenia). Statistical analyses were performed using Microsoft Excel 2019 (Microsoft, Redmond, Washington, DC, USA).

For statistical evaluation, we calculated proportions of TiO_2_-containing foods in different food (sub)categories. Additionally, we calculated the within-category proportion of foods containing TiO_2_, which was corrected with product market shares using the previously described sale-weighting approach [47]. In the investigated food categories, market share data were available for 59.8% (*N* = 3597) and 54.2% of foods (*N* = 3597) for 2017 and 2020, respectively. Sale-weighted proportions of TiO_2_-containing foods were calculated for each (sub)category separately, using the EAN barcode as a unique product identifier, with consideration of product packaging quantity and number of sold products in a 12-month period (based on nationwide sales data provided by food retailers). Food subcategories with less than four TiO_2_-containing foods were excluded from this analysis.

Descriptive analysis was used for proportions of food that contained TiO_2_, and the 95% confidence interval (95% CI) was calculated employing the Wilson score interval [48]. A two-tailed *z*-test was used to identify differences in the use of TiO_2_ between 2017 and 2020. The level of significance was set at *p* < 0.05. The following subcategories were excluded from this part of the analysis due to their low sample size of foods containing TiO_2_: processed fish products; canned fish with vegetable; sugar; ice cream and edible ices; desserts; flavored yogurt; cordials; soup; biscuits; side dishes and spreads and processed cheese.

## 3. Results and Discussion

**Figure 1:**
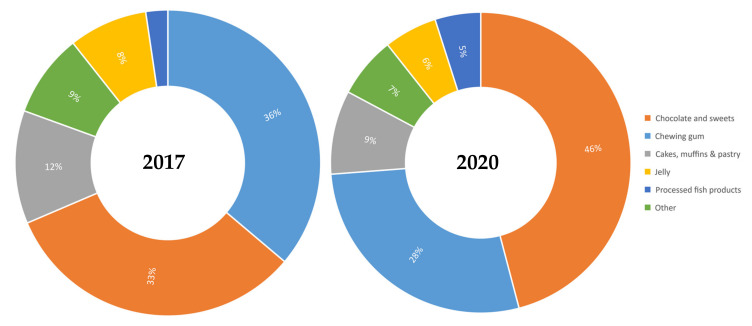
Distribution of foods containing TiO_2_ per food (sub)category in 2017 versus 2020.

The study was conducted on a sample of 6012 foods and beverages in 2017, and 6632 foods and beverages in 2020. Within the 15 selected food subcategories, 13 categories contained TiO_2_ in 2017 (215 products), and 10 categories in 2020 (122 products). In 2017, foods containing the highest amount of TiO_2_ were distributed in the chewing gum category, accounting for more than a third (36.3%) of the total amount of TiO_2_-containing foods (Figure 1). The second third was represented by chocolates and sweets (32.6%), followed by cakes, muffins and pastry (11.6%), jelly (8.4%) and processed fish products (2.3%). In 2020, almost half of TiO_2_ was distributed in the chocolate and sweets category (45.9%) and one third in the chewing gum category (27.9%), followed by cakes, muffins and pastry (9.0%), jelly (5.7%) and processed fish products (4.9%) (Figure 1). The remaining categories (each with less than a 3% share) represented 9% and 7% of TiO_2_-containing foods in 2017 and 2020, respectively (Figure 1, “Other”).

To provide insights into food reformulation practices, we also compared the composition of foods, which were found in both 2017 and 2020 dataset. Food matching using EAN barcodes resulted in 10,034 foods available in both datasets. Altogether, 88 of these products contained TiO_2_ in the 2017 sample, while in 2020 the use of TiO_2_ was retained in 49 products (55.7%). This indicates that food reformulation (removal of TiO_2_) was observed in 44.3% (*N* = 39) products.

Furthermore, we calculated per-category proportions of TiO_2_-containing foods in the food supply for both 2017 and 2020 (Table 1). For each year, we calculated the (nonweighted) proportion as a percentage of TiO_2_-containing foods of all available foods in the category. To gain an insight into the availability of such foods with a consideration of market share, we further employed the sale-weighting approach using nationwide 12-month sales data, provided by the largest food retailers in Slovenia. Such an approach provided information on whether TiO_2_ was used in market-leading brands or mostly in niche products. It should be noted that sales data were available for most, but not all foods in our study sample (see Section 2.1 for details). Missing data mostly reflect availability in discounter retailers.

Per-category, nonweighted proportions of TiO_2_-containing foods represented up to 70.3% in 2017 (Table 1). In 2017, the largest share of TiO_2_-containing foods was represented by chewing gum, comprising more than two third of the sample (70.3%), followed by jelly (9.7%) and processed fish products (7.0%) (Table 1). Chewing gum was also the highest ranked category (24.6%) in 2020, followed by processed fish products (6.9%) and jelly (4.4%).

In 2017, the sale-weighted proportion of TiO_2_-containing chewing gums was higher than the nonweighted proportion (85.5% vs. 70.3%), showing that this food additive was present in major brands. The situation changed considerably in 2020, when the sale-weighted proportion was much lower (3.1% vs. 24.6%). This indicates that a decrease in the use of TiO_2_ was even more pronounced in the best-selling products. We also compared the composition of the chewing gums, which contained TiO_2_ in 2017, and were still marketed in 2020. Out of 44 such products, 25 (56.8%) no longer contained TiO_2_ in 2020. This indicates that TiO_2_ dropped not only because of the arrival of new (TiO_2_-free) products and removal of older (TiO_2_-containing) products from the market, but also because of the reformulation of the existing products. However, the differences between sale-weighted and nonweighted proportions in other food categories were expressed to a much lower extent. Beside chewing gums, food subcategories with the highest sale-weighted proportions of foods with TiO_2_ were jelly (14.8%) and processed fish products (19.3%) in 2017. Considerably high sale-weighted proportions were also observed in these two categories in 2020 (20.2% and 19.0%, respectively).

The overall comparison of the 2017 and 2020 data showed a significant (*p* < 0.01) decrease in the use of TiO_2_ as a food additive from 2017 to 2020. Across the 15 observed food subcategories, 3.6% foods contained TiO_2_ in 2017, and 1.8% in 2020. This change could be attributed to the availability of new evidence on the potential health risks of TiO_2_, and by concerns raised by national health authority agencies [27,29]. As health concerns were also raised by EFSA [31], it is expected that responsible food producers will remove it from their products, despite the fact that it has not yet been officially restricted from the EU food supply. A statistically significant decrease in the use of TiO_2_ was also observed in specific food categories where TiO_2_ was a relevant additive in 2017. Sale-weighted proportions showed a similar trend, with the exception of the abovementioned processed fish products and jelly.

To our knowledge, this is the first repeated cross-sectional study on the use of TiO_2_ in the food supply in which trends in the use of TiO_2_ in prepacked foods were investigated with consideration of market share data. Such methodology makes the study results particularly relevant for the assessment of public health risks. While this makes comparisons with other studies difficult, relevant comparisons can be performed without consideration of sale-weighting. Mintel’s Global New Products Database (GNPD) [49], which contains data of newly launched foods in different countries (but not Slovenia), was used in the recent safety assessment of TiO_2_ by EFSA [31]. For a more relevant comparison, we combined several of Mintel’s food subcategories [50]. The highest proportion of TiO_2_-containing foods was observed in chewing gums (39%), followed by pastilles, gums, jellies and chews (10%), cakes, pastries and desserts (4%); and chocolate and sweets (3%) [31]. The Mintel database cannot be considered as cross-sectional, as it only contains data on newly launched products on the market (and not the overall situation in the food supply, where some market-leading brands have a long history of availability). Nevertheless, it should be mentioned that a decreasing trend in the use of TiO_2_ in newly launched foods was also observed. Data are also available for the US, where TiO_2_ was most commonly used in nonchocolate candy (32%), followed by cupcakes and snack cakes (14%), cookies (8%), coated pretzels and trail mix (7%), baking decorations (6%), gum and mints (4%) and ice cream (2%). However, it was assumed that many other foods contain TiO_2_, because in the US market TiO_2_ can be considered as an exempt color that does not require explicit declaration on the ingredient statement [51].

Exposure to TiO_2_ largely depends on an individual’s dietary habits. Since TiO_2_ is mainly present in processed foods such as chewing gum, cakes, pastry and other sweets, children and young people are more likely to be more exposed to higher TiO_2_ intake. For the United Stated and United Kingdom population it has been calculated that children potentially consumed two to four times as much TiO_2_ per kg body weight as an adult [2]. Similar studies revealing that children consume higher amount of TiO_2_ were observed across Europe [52] in the German and [53] Dutch population [21,54], and among Chinese young people [55].

Given scrutiny from regulatory bodies, the food industry has been working on TiO_2_ alternatives for some years. Reformulation initiatives were also stimulated by various nongovernmental active groups. In the US, for example, the *As You Sow* group put pressure on the Dunkin’ brand, which then withdrew the use of TiO_2_ from their sugar powdered donuts [56]. However, replacing TiO_2_ across all applications is technologically very challenging, as TiO_2_ is not only an excellent whitening pigment but also very cost effective [51]. However, rice starches now offer clean label solutions that can help with reducing the chipping and cracking of coatings [51]. Avalanche, starch and mineral based white opacifier are the most common replacements for TiO_2_ in food applications [57].

The strength of the present study is in the use of two large nationally representative cross-sectional food composition datasets in combination with market shares. While such an approach was used in the past for the assessment of public health risks related to specific nutrients, such as salt [58] and sugar [47], we showed that it can also be employed for food additives. The limitation of the study is that the used dataset did not contain all available foods, and that sales data were not available for the whole dataset. However, we should mention that data collection included all major retailers with a nationwide network of food stores, and that sales data were available from retailers who are responsible for over 50% of the food market. Another limitation is that the data on the use of TiO_2_ were extracted from food labels, and not determined in a laboratory. However, regulations require the labeling of functional additives, and the laboratory analysis of thousands of foods is not a feasible option in food supply studies. We should also note that our study did not investigate certain groups of foods in which a higher use of coloring agents could be expected, such as food supplements and food additive products (i.e., foods sold directly to consumers which are intended for coloring), which are also available to consumers in food stores.

## 4. Conclusions

According to the results of our study, the availability of prepackaged food products in Slovenia has undergone several improvements regarding the use of TiO_2_ in certain food categories. This is particularly notable in the category of chewing gum, where a reformulation trend was also observed. In recent years, we have witnessed an increased regulatory scrutiny of TiO_2_ as a food additive. In other studies, this was reflected in a decline in new launches of foods containing TiO_2_, while this cross-sectional study also confirmed such an observation in a whole supply of processed foods in Slovenia. We observed that in the past, the category with the most common use of TiO_2_ was chewing gum. In 2017, approximately 70% of chewing gums contained TiO_2_, and these products presented over 85% of the market share (by weight). However, the situation changed drastically; in 2020, approximately 25% of chewing gums contained TiO_2_, accounting for only 3% of the market share. The other two food categories with a high use of TiO_2_ were jelly and processed fish products, while in other food categories, less than 3% of products contained TiO_2_. Considering the EFSA’s 2021 announcement of TiO_2_ no longer being safe to use, a further decrease in the use of this additive is expected despite the fact that it has not yet been officially removed from the list of authorized food additives in the EU. Specific food categories were identified (i.e., chocolate and sweets), in which product reformulation is needed, and official controls by authorities will be most relevant.

## Figures and Tables

**Table 1:** (Sub)category proportions of foods containing TiO_2_ (E171) as food additive in the food supply for 2017 and 2020 (Slovenia).

Food Category	2017				2020				*z*-Test Statistic for Proportions	
	Total *N*	Added TiO_2_ *N*	% (95% CI)	Sale-Weighted Proportion (%)	Total *N*	Added TiO_2_ *N*	% (95% CI)	Sale-Weighted Proportion (%)	Proportion Change (95% CI)	*p*-Value
Chewing gum	111	78	70.3 (61.8–78.8)	85.5	138	34	24.6 (17.4–31.8)	3.1	45.6 (34.5–56.8)	<0.01
Jelly	185	18	9.7 (5.5–14.0)	14.8	159	7	4.4 (1.2–7.6)	20.2	5.3 (0.0–10.6)	0.03
Processed fish products	71	5	7.0 (1.1–13.0)	19.3	87	6	6.9 (1.6–12.2)	19.0	0.1 (−7.8–8.1)	ns
Cakes, muffins and pastry	569	25	4.4 (2.7–6.1)	3.0	639	11	1.7 (0.7–2.7)	1.1	2.7 (0.7–4.6)	<0.01
Chocolate and sweets	1917	70	3.7 (2.9–4.5)	2.8	2173	56	2.6 (1.9–3.2)	1.1	1.1 (0.0–2.1)	0.02
Canned fish with vegetable	60	1	1.7 (0.3–8.9)	*	60	0				ns
Sugar	127	2	1.6 (0.4–5.6)	*	108	0				ns
Ice cream and edible ices	431	6	1.4 (0.3–2.5)	1.6	586	3	0.5 (0.0–1.1)	*	0.9 (−0.4–2.1)	ns
Desserts	207	2	1.0 (0.4–2.3)	*	298	0				ns
Flavored yogurt	419	3	0.7 (0.2–2.1)	*	386	0				ns
Cordials	179	1	0.6 (0.1–3.1)	*	190	0				ns
Soup	264	1	0.4 (0.1–2.1)	*	257	1	0.4 (0.1–2.2)	*	0.0 (−1.1–1.1)	ns
Biscuits	1035	3	0.3 (0.1–0.9)	*	1122	2	0.2 (0.1–0.6)	*	0.1 (−0.2–0.5)	ns
Side dishes	199	0			224	1	0.5 (0.1–2.5)	*		ns
Spreads and processed cheese	238	0			205	1	0.5 (0.1–2.7)	*		ns
Total	6012	215	3.6 (3.1–4.0)	na	6632	122	1.8 (1.5–2.2)	na	1.8 (1.1–2.3)	<0.01

Notes: Data presented for food categories with at least one product with TiO_2_ in either the 2017 or 2020 dataset. 95% CI: 95% confidence interval; *N*—number of all products; ns—not significant; na—not applicable; *—low sample size (sale-weighted proportions not calculated for subsamples with *N* < 4).

## Data Availability

The data presented in this study are available on request from the corresponding author.
